# Generation of an hiPSC-Derived Co-Culture System to Assess the Effects of Neuroinflammation on Blood–Brain Barrier Integrity

**DOI:** 10.3390/cells11030419

**Published:** 2022-01-26

**Authors:** Daniel Bull, Christophe Schweitzer, Colette Bichsel, Markus Britschgi, Simon Gutbier

**Affiliations:** 1 ARUK UCL Drug Discovery Institute, University College London, London WC1E 6BT, UK; daniel.bull.17@ucl.ac.uk; 2 Roche Pharma Research and Early Development, Neuroscience and Rare Diseases Discovery and Translational Area, Roche Innovation Center Basel, 4070 Basel, Switzerland; christophe.schweitzer@roche.com; 3 Roche Pharma Research and Early Development, Pharmaceutical Sciences and The Roche Institute for Translational Bioengineering, Roche Innovation Center Basel, 4070 Basel, Switzerland; colette.bichsel.cb1@roche.com; 4 Roche Pharma Research and Early Development, Therapeutic Modalities, Roche Innovation Center Basel, F. Hoffmann-La Roche Ltd., 4070 Basel, Switzerland

**Keywords:** microglia, brain microvascular endothelial cells (BMECs), neuroinflammation, blood–brain barrier (BBB) integrity, transendothelial electrical resistance (TEER), hiPSC co-culture, cytokine secretion, transwell, neurovascular unit (NVU)

## Abstract

The blood–brain barrier (BBB) regulates the interaction between the highly vulnerable central nervous system (CNS) and the peripheral parts of the body. Disruption of the BBB has been associated with multiple neurological disorders, in which immune pathways in microglia are suggested to play a key role. Currently, many in vitro BBB model systems lack a physiologically relevant microglia component in order to address questions related to the mechanism of BBB integrity or the transport of molecules between the periphery and the CNS. To bridge this gap, we redefined a serum-free medium in order to allow for the successful co-culturing of human inducible pluripotent stem cell (hiPSC)-derived microglia and hiPSC-derived brain microvascular endothelial-like cells (BMECs) without influencing barrier properties as assessed by electrical resistance. We demonstrate that hiPSC-derived microglia exposed to lipopolysaccharide (LPS) weaken the barrier integrity, which is associated with the secretion of several cytokines relevant in neuroinflammation. Consequently, here we provide a simplistic humanised BBB model of neuroinflammation that can be further extended (e.g., by addition of other cell types in a more complex 3D architecture) and applied for mechanistic studies and therapeutic compound profiling.

## 1. Introduction

The neurovascular unit (NVU) is composed of specialised brain microvascular endothelial cells (BMECs), pericytes, glial cells, neurons and extracellular matrix components that all participate in maintaining the central nervous system (CNS) microenvironment [1,2,3]. The blood–brain barrier (BBB) is a key component of the NVU that, under homeostatic conditions, tightly regulates the exchange of molecules and cells between the peripheral blood and the brain parenchyma [4]. In particular, it prevents the entry of unwanted substances into the CNS by means of the highly selective nature of BMEC efflux transporters, as well as a tight junction formation that limits paracellular permeability [5,6]. Disruption to BMEC integrity can result in certain components from the blood reaching the CNS in much larger quantities than under physiological conditions, potentially triggering detrimental immune activation within the brain. This phenomenon, often referred to as neuroinflammation, results in neuropathological alterations associated with astrogliosis, microgliosis, the upregulation of inflammatory factors in brain interstitial and cerebrospinal fluid, as well as an increase in infiltrating immune cells [7,8]. Neurological disorders such as Alzheimer’s disease (AD), Parkinson’s disease (PD) and multiple sclerosis (MS) present different degrees and appearances of neuroinflammation, yet it remains unclear to what extent the BBB is impaired and how immune activation in the brain contributes towards disease progression and severity, especially in age-related neurodegenerative disorders [6,7,9,10,11,12].

Within a normal healthy human brain, the role of microglia still remains elusive. Preclinical model systems have characterised microglia as playing a key role in CNS homeostasis, neurogenesis, formation and elimination of synapses, vascular development, as well as cognition [13,14,15]. The discovery of multiple immune-related risk genes in large population studies has resulted in microglia and other innate immune pathways entering the spotlight of pathomechanistic research in sporadic forms of neurodegeneration, specifically in AD and PD [7,16,17,18]. How the physiological function or the genetic disease risks related to microglia may affect BBB integrity still needs to be explored.

The previous multicellular in vitro models of BBB impairment typically focused on pericyte and astrocyte involvement [19]. Given the crucial role of microglia within neurodegenerative diseases, in vitro BBB models should be augmented by a physiologically or pathologically relevant microglial component in order to perform mechanistic studies. Recently, several protocols for human inducible pluripotent stem cell (hiPSC)-derived microglia have been developed [20,21]. Current literature shows that hiPSC-derived microglia express key microglial markers, making them potentially physiologically superior to any other cellular model to study microglia function [21,22,23]. Given the many different phenotypes microglia can acquire, and that these cells can rapidly transition between phenotypes influenced by their environment, there is no consensus on a unique proteomic, genetic or functional signature that is required in order for the in vitro hiPSC-derived microglia to be comparable to human microglia in the brain [20]. Furthermore, hiPSC-derived microglia have been shown to closely resemble foetal microglia, a technical hurdle, which may hamper recapitulating certain aspects of age-related neurodegeneration in a dish [20].

The recent advancement of hiPSC derived cultures has led to many protocols being developed for patient-derived NVU cells in order to model certain disease aspects [24]. hiPSC-derived BMEC models have been suggested to recapitulate tight junction formation far better than any other in vitro model and subsequently produce in vivo physiologically relevant trans-endothelial/epithelial electrical resistance (TEER) values >1000 Ω·cm^2^ [24,25]. Furthermore, the inclusion of hypoxia during the differentiation process has been suggested to enforce a more endothelial phenotype [26].

Here we describe a novel, simplistic humanised co-culture transwell system consisting of hiPSC-derived microglia and hiPSC-derived BMECs, that can be utilised for dissecting the mechanisms through which microglia regulate BBB function, therapeutic compound profiling, as well as a provide a foundation for which co-culture systems with a complex multicellular 3D architecture could be generated.

## 2. Materials and Methods

### 2.1. hiPSC Cultures 

All work with hiPSC and the derived cell types was performed under the respective Swiss legislation, ethical guidelines and approval. hiPS_SFC_086 (generated by the StemBANCC Innovative Medicines Initiative consortium [27]; https://www.imi.europa.eu/projects-results/project-factsheets/stembancc, accessed on 20 December 2021) and BIONi010-C (Bioneer, DK-2970 Hørsholm, Denmark) lines were cultured in mTeSR1 Plus (StemCell Technologies, Vancouver, BC, Canada) on 12.5 µg/mL rhLaminin-521 (BioLamina, Sundbyberg, Sweden) coated plates. RevitaCell (Gibco, Waltham, MA, USA) would be supplemented into the medium for splitting and thawing.

### 2.2. hiPSC-Derived BMECs

**Figure 1:**
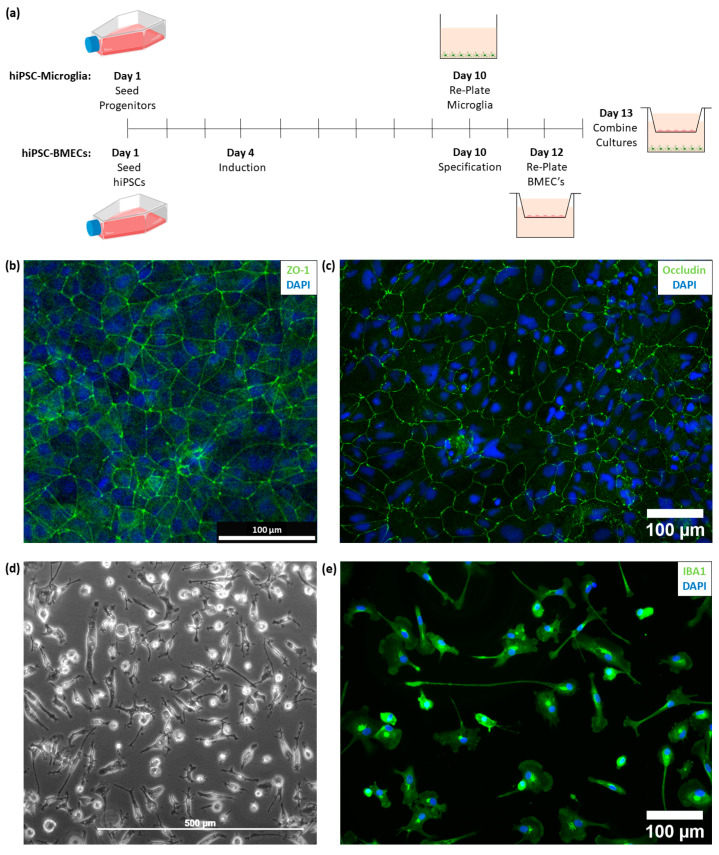
(**a**) Timeline for the differentiation of the human inducible pluripotent stem cell (hiPSC)-derived brain microvascular endothelial cells (BMECs) (pink, in the transwell insert) and hiPSC-derived microglia (green, at the bottom of the plate) cultures, as well as when to combine the cultures to generate the transwell co-culture system. Images were created with BioRender.com. (**b**) Immunofluorescence image of hiPSC-derived BMECs with zonula occludens-1 (ZO-1, green) and 4′,6-Diamidino-2-Phenylindole (DAPI, blue) staining. (**c**) Immunofluorescence image of hiPSC-derived BMECs with occludin (green) and DAPI (blue) staining. (**d**) Bright field image of the hiPSC-derived microglia cells during differentiation. (**e**) Immunofluorescence image of hiPSC-derived microglia with ionized calcium-binding adaptor molecule 1 (IBA1, green) and DAPI (blue) staining.

The differentiation protocol is summarised in Figure 1a and is based on the Park et al. manuscript [26]. Briefly, 5000 hiPS_SFC_086 cells/cm^2^ were seeded on growth factor reduced (GFR) Matrigel (Corning, NY, USA) coated flasks (Corning) in mTeSR1 Plus supplemented with RevitaCell. After 3 days of expansion, the medium on the cells was replaced with induction medium (Table S1) for 6 days in hypoxic (5% O_2_) conditions. The cells were then further expanded in specification medium (Table S2) for 2 days. On day 12, transwells (0.4 µm, 24 well, Greiner, Frickenhausen, Germany) were coated with 400 µg/mL of collagen IV (Sigma, St. Louis, MO, USA) and 100 µg/mL of fibronectin (Corning) for 2 h at 37 °C. After coating, the transwell membrane was washed once with 1x Dulbecco’s phosphate-buffered saline (DPBS, −/−, Gibco). hiPSC-derived BMECs were then detached with accutase (StemCell Technologies) and re-plated at 250,000 cells/transwell in specification medium (final volume of 250 µL in apical and 950 µL in the basolateral chambers).

### 2.3. Embryoid Body (EB) Formation, Plating and Maturation

EB formation was previously described in detail from the Gutbier et al. manuscript [28]. Briefly, BIONi010-C cells were seeded into AggreWell 800 (StemCell Technologies) plates in accordance with the manufactures instructions. The next day, induction was started by exchanging 75% of the mTeSR1 plus medium with mTeSR1 plus medium supplemented with 50 ng/mL recombinant human bone morphogenetic protein 4 (rhBMP4), 50 ng/mL recombinant human vascular endothelial growth factor (rhVEGF) and 20 ng/mL recombinant human stem cell factor (rhSCF) (Biotechne, Minneapolis, MN, USA). This process was repeated for the following 2 days. EB’s were then harvested and plated at a density of 1 EB/cm^2^ in GFR Matrigel-coated flasks within myeloid factory medium (Table S3). hiPSC-derived macrophage progenitors were collected from the supernatant by centrifugation.

### 2.4. Differentiation of hiPSC-Derived Macrophage Progenitors to hiPSC-Derived Microglia Cells

The differentiation protocol is summarised in Figure 1a and is described in detail from the Reich et al. manuscript [21]. Briefly, flasks and plates were pre-coated with 10 µg/mL of fibronectin for 3 h at room temperature before washing three times with water. hiPSC-derived macrophage progenitors were seeded into a pre-coated flask in microglia differentiation medium (Table S4) at a density of 50,000 cells/cm^2^. Half the medium was replaced every 2 days for a total of 10 days. On day 10, the cells were detached with accutase and collected by centrifugation before being re-plated at the same density onto fibronectin-coated 24 well plates in microglia differentiation medium (Table S4). The cells were cultured in a standard incubator (20% O_2_) throughout.

### 2.5. TEER Readout

The TEER between the apical and basolateral chambers was measured using an EVOM3 Volt/Ohm meter combined with an STX2-Plus electrode (World Precision Instruments, Sarasota, FL, USA). Samples were prepared in triplicate, and the average values were calculated. Blank values were subtracted from the experimental measurements, and Ohm (Ω) measurements were corrected for transwell filter area.

### 2.6. Effect of Microglial Differentiation Cytokines on Barrier Integrity

On day 13 of the BMEC differentiation protocol, the medium on the hiPSC-derived BMECs was replaced with basal medium (RPMI 1640 and 10 U/mL penicillin-streptomycin (P/S)) supplemented with different combinations of recombinant human transforming growth factor-beta 1 (rhTGF-β1), recombinant human interleukin-34 (rhIL-34), and/or recombinant human macrophage-colony stimulating factor (rhM-CSF) (Table S4). Then, 24 h later, the TEER was measured.

### 2.7. Comparison of Different Culture Media on Barrier Integrity

On day 13 of the BMEC differentiation protocol, the medium on the hiPSC-derived BMECs was replaced with either human endothelial medium (human endothelial serum-free medium (Gibco), 1% fetal bovine serum (FBS, Gibco) and 10 U/mL P/S (Gibco)) or microglia medium without rhTGF-β1 supplementation (Table S5). Then, 24 h later, the TEER was measured.

### 2.8. Effect of hiPSC-Derived Microglia on Barrier Integrity When Exposed to LPS

On day 13, the hiPSC-derived BMECs and hiPSC-derived microglia cell cultures were combined (Figure 1a), and the medium in both chambers was replaced with co-culture medium (Table S5). Then, 24 h later, 100 uL of medium was removed from the basolateral compartments, and 100 uL of RPMI 1640 supplemented with lipopolysaccharides from Escherichia coli O55:B5 (LPS, Sigma, L5418, Lot:117M4183V) was added to the basolateral compartment to form a total concentration of either 0, 1, 10 or 100 ng/mL of LPS (950 µL total volume). The TEER was measured every 3, 6, 9 and 24 h. Bar graph plots are representative of the percentage change in TEER between the 0 h and 9 h timepoints.

### 2.9. Cytokine Quantification Assay

Pre-LPS addition, 100 uL of medium was removed from the basolateral compartments. After 9 h of LPS exposure, 100 uL of medium was also removed from the basolateral compartments. All samples were immediately frozen. Cytokine quantification of tumour necrosis factor-alpha (TNF-α), interleukin 6 (IL-6), interleukin 8 (IL-8), chemokine (C-X-C motif) ligand 1/growth-regulated oncogene alpha (CXCL1/GROα), interleukin 10 (IL-10), chemokine (C-C motif) ligand 2/monocyte chemoattractant protein 1 (CCL2/MCP-1), chemokine (C-X-C motif) ligand 2/growth-regulated oncogene beta (CXCL2/GROβ), interleukin 1 beta (IL-1β), chemokine (C-C motif) ligand 3/macrophage inflammatory protein 1 alpha (CCL3/MIP-1α), chemokine (C-C motif) ligand 5/regulated on activation, normal T cell expressed and secreted (CCL5/RANTES), chemokine (C-C motif) ligands 4/macrophage inflammatory protein 1 beta (CCL4/MIP-1β), interleukin 1 alpha (IL-1α), interleukin 4 (IL-4), interleukin 13 (IL-13), chemokine (C-X-C motif) ligand 5 (CXCL5) and interleukin 12 p70 (IL-12 p70) was performed through the use of a Luminex^®^ cytokine assay (Bio-Techne) in accordance with the manufacturer’s instructions. Experimental values are representative of the cytokine concentration change between 0 h and 9 h of LPS exposure. More details about the measurement procedure and calculation are provided in Tables S6 and S7.

### 2.10. Immunocytochemsitry

BMECs and microglia were fixed in 4% paraformaldehyde (electron microscopy services, Hatfield, PA, USA) for 20 min before being washed three times with 1x DPBS. Cells were blocked with a solution consisting of 10% goat serum (Abcam, Cambridge, UK) and 0.3% Tween 20 (Roche, Basel, Switzerland), diluted in 1x DPBS for 1 h at room temperature. Zonula occludens-1 (ZO-1, also known as tight junction protein-1) primary antibody (1:100, Invitrogen) or occludin primary antibody (1:100, Thermo Fisher Scientific, Waltham, MA, USA) was diluted in blocking solution and added to the fixed BMECs overnight at 4 °C. Ionized calcium-binding adaptor molecule 1 (IBA1, 1:200, Wako, Osaka, Japan) was diluted in blocking solution and added to the fixed microglia cells overnight at 4 °C. The following day, the cells were washed three times with 1x DPBS before a solution of goat anti-mouse or anti-rabbit 488 Alexa Fluor secondary antibody (1:500, Invitrogen) and 4′,6-Diamidino-2-Phenylindole (DAPI, 1:1000, Invitrogen, Waltham, MA, USA), diluted in blocking solution, was added to the cells for 1 h at room temperature in the dark. After incubation, the cells were washed a further three times with 1x DPBS. For BMECs cultured on transwells, the transwell membrane was cut from the transwell and placed onto a slide for imaging. All imaging was performed on the SP8 lightning confocal microscope (Leica, Wetzlar, Germany).

### 2.11. Statistics

Results are expressed as mean ± standard deviation. All experiments were performed three times (biological replicates), with at least three technical replicates per condition and for each biological replicate. Statistical methods for analysing the various data sets are indicated directly in the figure legends. Data were analysed using Graphpad Prism 9 (GraphPad Software version 9.3.1, GraphPad, San Diego, CA, USA).

## 3. Results

### 3.1. Development of the hiPSC-Derived BMEC and hiPSC-Derived Microglia Co-Culture System

hiPSC-derived macrophage progenitors were seeded and differentiated for a total of 13 days to form hiPSC-derived microglia, with the cells being re-plated on day 10 into a 24 well plate (Figure 1a). In parallel, hiPSCs were seeded and expanded before being induced under hypoxic conditions. These cells were then subjected to retinoic acid and fibroblast growth factor specification to generate hiPSC-derived BMECs, before being re-plated on day 12 into a transwell insert. Both hiPSC-derived monocultures were combined on day 13 in a co-culture medium (Table S5). We show that the hiPSC-derived BMECs express zonula occludens-1 (ZO-1, also known as tight junction protein-1) and occludin (Figure 1b,c), and that the hiPSC-derived microglia express ionized calcium-binding adaptor molecule 1 (IBA1), as well as display the relevant morphology (Figure 1d,e).

### 3.2. TGF-β1 Alters hiPSC-Derived BMEC Barrier Integrity

**Figure 2:**
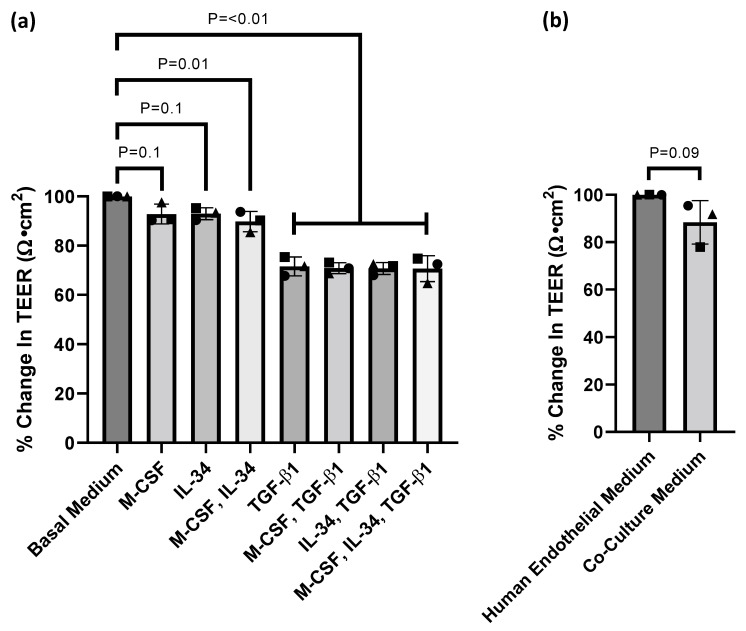
Recombinant human transforming growth factor-beta 1 (rhTGF-β1) strongly diminishes hiPSC-derived BMEC barrier properties. (**a**) On day 13 of the differentiation process, basal medium supplemented with ±100 ng/mL recombinant human interleukin-34 (rhIL-34), ±25 ng/mL recombinant human macrophage-colony stimulating factor (rhM-CSF) and ±50 ng/mL rhTGF-β1 was added to the BMECs with the TEER measured 24 h later. The percentage change in trans-endothelial electrical resistance (TEER) was normalised to the basal medium experimental condition (One-way ANOVA with Dunnett’s multiple comparisons test, *p* values displayed on the graph, *n* = 3). (**b**) On day 13, the medium on the hiPSC-derived BMECs was replaced with either human endothelial medium or co-culture medium (Table S5), and the TEER was measured 24 h later. The percentage change in TEER was normalised to the human endothelial medium experimental condition (two-tailed unpaired *t*-test, *p* values displayed on the graph, *n* = 3). Each graphical symbol shape (square, triangle and circle) represents each set of experimental replicates.

A big challenge of combining different cell types in a co-culture system is the identification of growth conditions that are the best compromise for the cell types used. The goal of our experiments was to develop culture conditions that allow for the homeostatic co-culture of hiPSC-derived BMECs and hiPSC-derived microglia. Towards the end of the differentiation protocol, hiPSC-derived BMECs are typically cultured in a serum-containing medium [24]. However, this would strongly influence the serum-free cultured hiPSC-derived microglia and potentially prohibit a physiologically relevant state. Therefore, using TEER, we wanted to assess whether hiPSC-derived microglia differentiation medium affects the hiPSC-derived BMEC barrier properties. hiPSC-derived BMECs in basal medium alone reached an experimental TEER value ranging from 2749–3352 Ω·cm^2^. Our results show that supplementation of rhTGF-β1 into the culture medium resulted in a robust 30% attenuation of the TEER value compared to the basal medium, whereas rhM-CSF and rhIL-34 supplementation showed only a minor effect (Figure 2a). As rhTGF-β1 supplementation results in a substantial reduction in barrier integrity, we omitted it in the final culture medium as declining TEER values would influence experimental outcome. 

In the next step of optimisation, we wanted to determine how the modified serum-free microglia medium (Table S5) compared to the classical human endothelial serum-containing medium in order to assess if the modified microglia medium had any effect on BMEC barrier integrity, measured by TEER. hiPSC-derived BMECs in human endothelial medium alone reached an experimental TEER value ranging from 2399–3846 Ω·cm^2^. Overall, only a minor effect in the TEER was observed between the two different medium compositions (Figure 2b). The modified serum-free microglia medium would now be referred to as the co-culture medium.

### 3.3. hiPSC-Derived Microglia Exposed to LPS Disrupt Barrier Integrity in a Co-Cultured System

Having established that barrier integrity of hiPSC-derived BMECs remains largely intact in the presence of the co-culture medium, we next established a co-culture of the two hiPSC-derived cell types in the same well of a culture plate: hiPSC-derived microglia were cultured adherently at the bottom of the basolateral compartment while hiPSC-derived BMEC were kept on the apical side of a membrane separating the two compartments. Both cell types were combined in the same well on day 13 of differentiation (Figure 1a).

**Figure 3:**
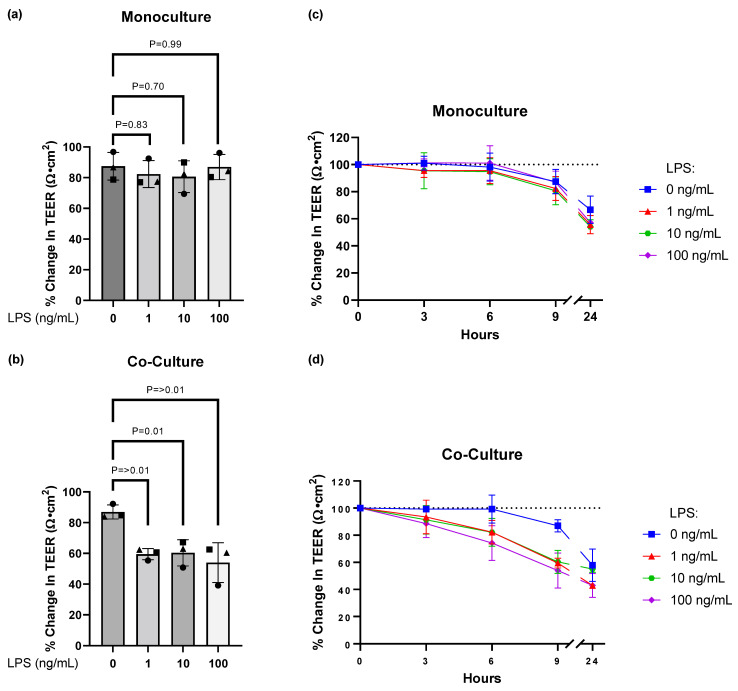
hiPSC-derived microglia exposed to lipopolysaccharides (LPS) disrupts hiPSC-derived BMEC barrier integrity. (**a**) On day 14, 0, 1, 10 or 100 ng/mL of LPS was added to the basolateral compartment of hiPSC-derived BMECs (monoculture), and the percentage change in TEER between 0 h and 9 h calculated (0 h TEER baseline: 2395–3741 Ω·cm^2^). Statistical comparisons were performed against the 0 ng/mL LPS monoculture experimental condition (One-way ANOVA with Dunnett’s multiple comparisons test, *p* values displayed on the graph. (**b**) On day 13 of the differentiation process, both hiPSC-derived cultures were combined to form the co-culture system. Then, 24 h later, 0, 1, 10 or 100 ng/mL of LPS was added to the basolateral compartment and the percentage change in TEER between 0 h and 9 h calculated (0 h TEER baseline: 2134–3656 Ω·cm^2^). Statistical comparisons were performed against the 0 ng/mL LPS co-culture experimental condition (One-way ANOVA with Dunnett’s multiple comparisons test, *p* values displayed on the graph. (**c**) The time-course of the percentage change in TEER observed for the hiPSC-derived BMECs (monoculture) upon LPS (0, 1, 10 or 100 ng/mL) exposure. (**d**) The time-course of the percentage change in TEER observed for the hiPSC-derived co-culture system upon LPS (0, 1, 10 or 100 ng/mL) exposure. In (**a**,**b**), we performed the experiment three times (three biological replicates), and in each experiment, we have included three technical replicates per condition. Each symbol in a condition (square, triangle and circle) indicates a separate experiment, and the bar graph illustrates the mean ± standard deviation of the three experiments. In (**c**,**d**) we performed three time-course experiments with four different conditions, and we included at least three technical replicates per condition. At each timepoint, the mean ± standard deviation of the three biological replicates is shown for each condition.

LPS is a bacterial component that triggers an inflammatory phenotype in cultured microglia, and it is used to mimic an experimental neuroinflammatory environment [29]. In order to reduce the potential direct effect of LPS on the hiPSC-derived BMECs, LPS was added to the microglia in the basolateral (abluminal) compartment for 9 h on day 14. LPS exposure to the hiPSC-derived BMEC monoculture had no effect on barrier integrity (Figure 3a). However, in a co-culture setting, hiPSC-derived microglia exposed to LPS disrupted the barrier properties of the hiPSC-derived BMECs by 40–50%, as shown by a decrease in the experimental TEER (Figure 3b). Originally, we tested different timepoints over 24 h to measure the effects of LPS on TEER (Figure 3c,d). The 9 h timepoint demonstrated to be the most optimal in order to detect a robust effect of LPS, as the BMECs in both a monoculture and co-culture setting appear to deteriorate in their barrier integrity at 24 h.

### 3.4. hiPSC-Derived Microglia Exposed to LPS Results in Secretion of Multiple Potential Barrier Altercating Cytokines

**Figure 4:**
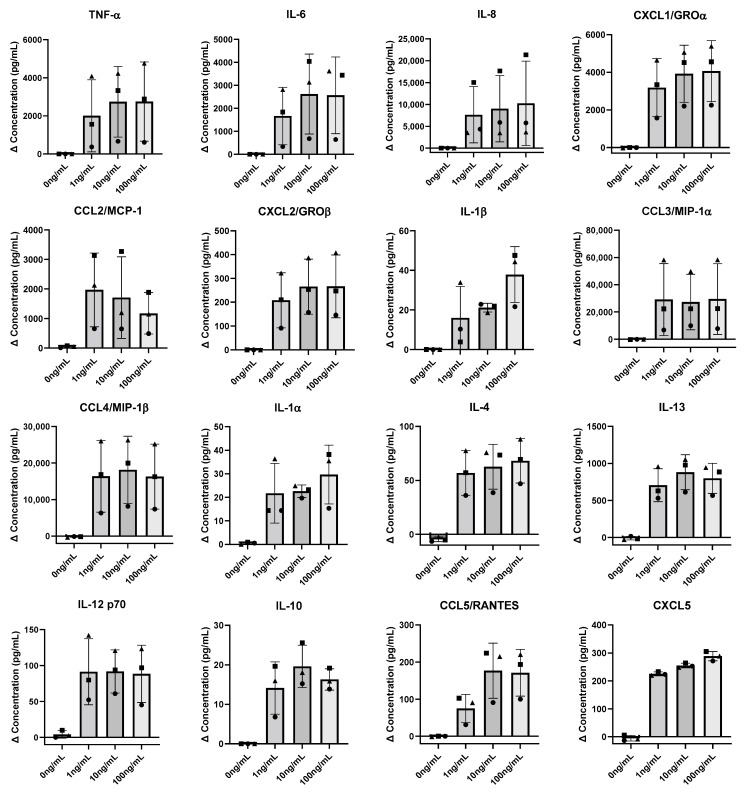
hiPSC-derived microglia exposed to LPS secrete elevated levels of cytokines. After 0 and 9 h of LPS (0, 1, 10, 100 ng/mL) exposure in the co-culture system, cell culture supernatant was removed and cytokine concentration quantified with a Luminex^®^ multiplex assay. Data displays the mean change in cytokine concentrations between 0 h and 9 h of LPS exposure. Each graphical symbol shape (square, triangle and circle) represents each set of biological replicates with at least three technical replicates. Mean values ± standard deviation of cytokine concentrations for the 0 h and 9 h timepoints are provided in Table S6.

It has been well documented that LPS exposed microglia secrete multiple cytokines that may contribute to BBB disruption [22,29,30]. In order to explore the association of the experimental TEER results with a potential cytokine release by the microglia, cell culture supernatant was removed from the basolateral compartment pre- and post-LPS exposure, followed by quantification of a panel of sixteen cytokines that have previously been associated with neuroinflammation [22,29,30]. We show that all cytokine levels were elevated when LPS was added to the hiPSC-derived microglia relative to cells that were not exposed to LPS (Figure 4 and Table S6). Cytokine secretion was demonstrated to be microglia dependent as the secretion levels of the hiPSC-derived BMEC monoculture were far below that of the co-culture values (Figure S1 and Table S7).

## 4. Discussion

Modelling the BBB in vitro remains a challenge due to its in vivo multicellular 3D architecture and the intricate relationship between the cells of the BBB, the periphery and the brain. In addition, the BBB manifests different properties and appearances in the different brain regions and is highly influenced by pathological changes in the brain and the periphery, including neuroinflammatory processes in neurodegeneration [12]. Stepwise approaches to add the different cell types (typically primary and iPSC-derived) and mimicking their interactions is currently being explored with the goal to build more complete brain organoid cultures for research, as well as industrial drug screens and mechanistic testing [30]. Here we show data from a transwell-based co-culture system between hiPSC-derived BMECs and hiPSC-derived microglia in an attempt to explore the interplay between these two cell types in an experimental inflammatory context.

Development and thorough characterisation of both the hiPSC-derived BMECs and hiPSC-derived microglia cells is reported by Park et al. and Reich et al. [21,26]. Building on this, we show that the hiPSC-derived BMECs express ZO-1 and occludin (Figure 1b,c) and that hiPSC-derived microglia express IBA1, as well as display the relevant morphology (Figure 1c,d). Using the same differentiation methodology, we previously demonstrated that in a monoculture setting, the hiPSC-derived microglia exhibit a disease-associated (DAM)-‘like’ gene expression, making them a potentially physiologically relevant microglial model to model a disease-phenotype including neuroinflammation [21].

Here, we redefined the microglia differentiation medium to allow for the successful co-culturing of hiPSC-derived microglia with hiPSC-derived BMECs, with minimal influence on barrier integrity, shown by the TEER values (Figure 2b). We demonstrated that rhTGF-β1 supplementation would need to be avoided from the co-culture medium in order to retain endothelial barrier integrity (Figure 2a). TGF-β1 promotes endothelial to mesenchymal transition (EndMT), whereby endothelial cells lose polarity and increase migration [31,32]. TGF-β1 has also been involved in inducing endothelial apoptosis and angiogenesis [33,34,35]. Together, these observations suggest that TGF-β1 induces a loss of the quiescent phenotype and explains the observed substantial drop in TEER values.

Although TGF-β1 is crucial for microglial homeostasis, it has been demonstrated that rhTGF-β1 supplementation is not important for mature hiPSC derived-microglial survival in vitro [36]. Furthermore, the silencing of TGF-β1 signalling in mature microglia does not appear to affect the homeostatic microglia gene expression signature [37]. However, it is still unclear what impact omitting rhTGF-β1 from the medium for 24 h (day 13) has on the overall microglial phenotype. We speculate that this medium composition (Table S5) will allow for the successful integration of hiPSC derived-microglial into established BBB models in order to provide more physiological relevance. 

We further demonstrate that hiPSC-derived microglia exposed to LPS causes a robust reduction of the hiPSC-derived BMEC TEER, even at low concentrations (Figure 3b). Park et al. showed that in order to sustain barrier function over time, medium flow is needed [26]. In our static transwell system, the lack of shear stress means that the TEER of the differentiated hiPSC-derived BMECs decreases over time (Figure 3c,d). Therefore, longer exposure timepoints such as 24 h could not be used as the BMEC monoculture TEER values began to deteriorate (Figure 3c,d). Therefore, a 9 h timepoint was selected as that is when the greatest difference in TEER was observed for the LPS exposed conditions in the co-culture system (Figure 3d). Perhaps future experiments could address whether a declining TEER can be minimised by switching the co-culture medium in the apical chamber with a serum-supplemented human endothelial medium. Mimicking the physiological location of serum components in the apical (and thus vascular luminal side) may help better maintain the BMEC TEER so that the LPS mediated effects can be observed for a greater period of time. It is worth noting that we added LPS to the basolateral compartment in order to achieve reliable stimulation of the microglia, as well as minimise any LPS mediated effects on the hiPSC-derived BMECs. 

Our results also display a lack of an LPS concentration-dependent effect, indicating that the chosen dose range has probably already reached a plateau in modulating the very sensitive hiPSC-derived microglia (Figure 3b). In the context of an experimental in vivo model of systemic inflammation induced by LPS, it was proposed that microglia can be stimulated to migrate towards the cerebral vasculature and initially help to maintain the BBB before sustained inflammation impairs BBB function [38]. Our in vitro setup without physical contact between the hiPSC-derived BMECs and microglia may replicate the detrimental part of the mentioned in vivo experiment and indicate that soluble factors alone can influence the BBB integrity in vitro. Therefore, testing different culture conditions (including physical contact between the cells) and exposure times may help in mimicking early aspects of microglia-BMEC interactions.

To better understand the relationship between LPS exposed hiPSC-derived microglia and the subsequent reduction in hiPSC-derived BMEC TEER, we showed that LPS treated microglia produced strongly elevated levels of cytokines (Figure 4). The measured cytokines and chemokines TNF-α, CXCL10, CCL2, IL-6 and IL-1β have all been associated with BBB disruption, whereas CXCL1, CXCL2, CCL3 and CCL5 have been reported to be elevated at the time of BBB disruption, and together resulting in the infiltration of T cells and monocytes into the CNS, further accelerating BBB disturbance [39,40,41,42,43,44]. Our data generated in the static in vitro system shows that under an inflammatory insult such as LPS, hiPSC-derived microglia, and not the hiPSC-derived BMECs, contribute largely to the secretion of cytokines and chemokines reported in studies that are associated with neuroinflammatory events (Figure 4 and Figure S1). Based on these results, we speculate that future experiments must analyse physical cell-cell contact in the more complex 3D architecture cellular systems in order to better understand microglia’s role within neuroinflammation.

Similar to our TEER data, cytokine release was not modulated by the different LPS concentrations (Figure 4). Perhaps these cytokine results explain why the TEER was similar amongst the different LPS concentrations (Figure 3b). The LPS stimulus is known to elicit a strong neuroinflammatory response [29]. However, given that microglia in the normal CNS will not likely be subject to LPS, perhaps stimuli such as dying neurons or amyloid plaque-like features could be used in order to address questions related to neuropathological changes observed in neurodegenerative disease disorders. Considerable variation was also observed between our experimental cytokine repeats (Figure 4). This is most likely due to the variability in microglia re-plating numbers (day 10) and the transcriptional differences in the harvest age of the hiPSC-derived microglial cells [45]. Hence stringent technical optimisation, as well as quality control processes for the production of all hiPSC-derived cell types, will be key to establishing standardised protocols towards consistent culture conditions for research, drug screening and testing.

The main limitation of our cellular model is the epithelial characteristics of the hiPSC-derived BMECs [46,47]. A recent transcriptomic meta-analysis has shown hiPSC-derived BMECs to express epithelial genes, as well as possess an inadequate expression of genes related to vascular lineage [46,48]. Although we did not perform our own in-depth characterisation of this cell type, current literature suggests that the hiPSC-derived BMECs exhibit both epithelial and endothelial characteristics [47]. However, hiPSC-derived BMECs have been suggested to be the only available BBB model possessing both high passive barrier and functional transporter characteristics that can serve as a reasonable model of the in vivo human BBB [47]. Shear stress, the overexpression of endothelial transcription factors, as well as aggregate architecture, have all been demonstrated to improve vasculature features [47,49,50]. The implementation of these techniques into the differentiation protocol could perhaps be utilised in order to push the hiPSC-derived BMECs closer towards an endothelial phenotype. Furthermore, given the epithelial/endothelial fluidity of our model perhaps hiPSC-derived macrophages could be incorporated to better address questions related to gut inflammation [49].

In order to progress our model towards a more physiologically relevant BBB system, additional hiPSC-derived NVU cell types should be included. It has been reported that the co-culturing of hiPSC-derived BMECs with pericytes, astrocytes and/or neurons significantly elevates the TEER [50,51]. However, integration of these extra cell types needs to be thoroughly characterised as medium components could drastically affect cell survival and activation, influencing experimental outcome and interpretation.

As touched on previously, the transwell lacks physiological relevance due to the lack of shear stress and limited cell-cell contact [19]. In order to address these issues, we could see our model being repurposed to a microfluidic format. In this small scale arrangement, compound permeability studies could be implemented in order to develop therapeutic treatments targeting microglia to dull the neuroinflammatory response and thus prevent BBB disruption.

Overall, we redefined a serum-free medium in order to allow for the successful co-culturing of hiPSC-derived microglia and hiPSC-derived BMECs, without influencing barrier properties as measured by TEER. Our medium composition can be used in order to allow for the successful introduction of hiPSC-derived microglia into established BBB models. We further demonstrate that hiPSC-derived microglia exposed to LPS weakens barrier integrity, possibly through the secretion of several cytokines and additional factors, supporting the evidence that microglia may play a key role in mediating BBB disruption a hallmark of many neurological disorders. We propose that an optimised version of our humanised system can be utilised to provide proof-of-concept for neurodegenerative stressors or can be employed for mechanistic studies related to therapeutic compounds, including transport and mode of action.

## Data Availability

Data presented in this paper can be requested from the corresponding authors.

## Supplementary Materials

The following are available online at https://www.mdpi.com/article/10.3390/cells11030419/s1, Figure S1: Cytokine secretion from LPS treated hiPSC-derived BMECs. Table S1: Induction medium composition. Table S2: Specification medium composition. Table S3: Factory medium composition. Table S4: Microglia medium composition. Table S5: Co-culture medium composition. Table S6: Co-culture cytokine data. Table S7: BMEC monoculture cytokine data.
