# Supplementary material for: Generation of an hiPSC-Derived Co-Culture System to Assess the Effects of Neuroinflammation on Blood–Brain Barrier Integrity
Source: Cells. 2022 Jan 26;11(3):419. doi: 10.3390/cells11030419 (PMC8834542; doi:10.3390/cells11030419)
Supplement: Supplementary file 1 [file cells-11-00419-s001.zip › cells-1546817-supplementary.pdf]

Supplementary Information

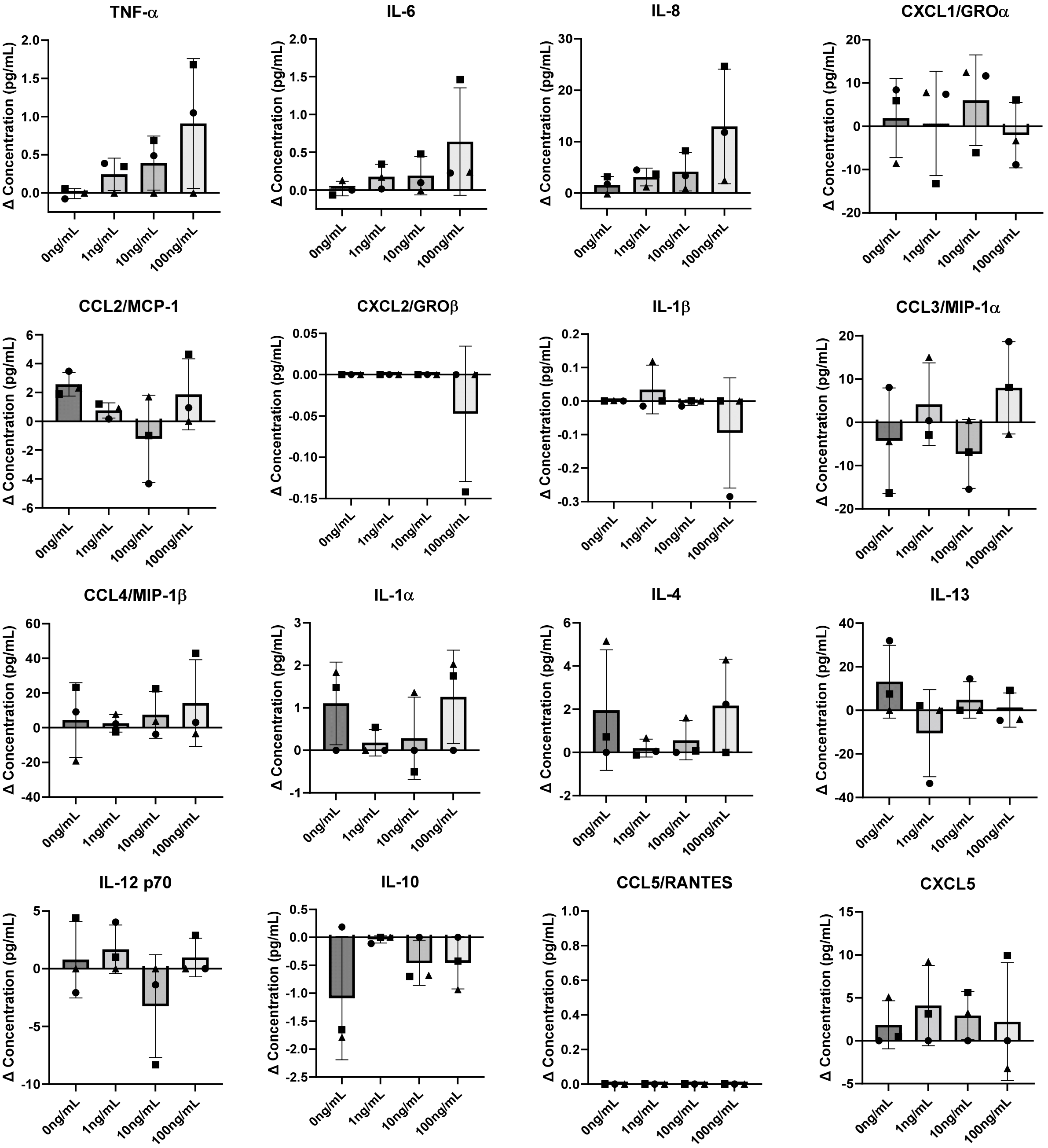

Figure S1. hiPSC-derived BMECs exposed to LPS secrete only very low levels of cytokines in comparison to the co-culture system. After 0 h and 9 h of LPS (0, 1, 10, 100 ng/mL) exposure in the BMEC (monoculture) system, cell culture supernatant was removed and cytokine concentration quantified with a Luminex® multiplex assay. Data displays the mean change in cytokine concentrations between 0 h and 9 h of LPS exposure. Each graphical symbol shape (square, triangle and circle) represents each set of biological replicates with at least three technical replicates. Mean values ± standard deviation of cytokine concentrations for the 0 h and 9 h timepoints are provided in Table S7.

Table S1. Induction Medium Composition

Reagent	Catalogue Number	Manufacture	Final Concentration
DMEM/F12	21331020	Gibco	-
KOSR	10828028	Gibco	20%
MEM NEAA	11140035	Gibco	1X
GlutaMAX	35050061	Gibco	1 mM
β-Mercaptoethanol	31350010	Gibco	0.1 mM
Penicillin-Streptomycin	15140148	Gibco	10 U/mL

Table S2. Specification Medium Composition

Reagent	Catalogue Number	Manufacture	Final Concentration
Human Endothelial SFM	11111044	Gibco	-
FBS	A3160502	Gibco	1%
Penicillin-Streptomycin	15140148	Gibco	10 U/mL
rhFGF	100-18B	Peprotech	20 ng/mL
Retinoic Acid	R2625	Sigma	10 uM

Table S3. Myeloid Factory Medium Composition

Reagent	Catalogue Number	Manufacture	Final Concentration
X-VIVO 15	BE02-060Q	Lonza	-
GlutaMAX	35050061	Gibco	2 mM
Penicillin-Streptomycin	15140148	Gibco	10 U/mL
β-Mercaptoethanol	31350010	Gibco	50 μM
rhMCSF	130-096-493	Miltenyi Biotec	100 ng/mL
rhIL-3	130-093-909	Miltenyi Biotec	25 ng/mL

Table S4. Microglia Medium Composition

Reagent	Catalogue Number	Manufacture	Final Concentration
RPMI 1640 + Glutamax	61870-010	Gibco	-
Penicillin-Streptomycin	15140148	Gibco	10 U/mL
rhIL-34	130-108-977	Miltenyi Biotec	100 ng/mL
rhMCSF	130-096-493	Miltenyi Biotec	25 ng/mL
rhTGF-β1	130-108-969	Miltenyi Biotec	50 ng/mL

Table S5. Co-Culture Medium Composition

Reagent	Catalogue Number	Manufacture	Final Concentration
RPMI 1640 + Glutamax	61870-010	Gibco	-
Penicillin-Streptomycin	15140148	Gibco	10 U/mL
rhIL-34	130-108-977	Miltenyi Biotec	100 ng/mL
rhMCSF	130-096-493	Miltenyi Biotec	25 ng/mL

Table S6. Cytokine concentrations (pg/mL) at 0 h and 9 h used to calculate the delta mean values provided in Figure 4 (co-culture system exposed to LPS). Each value below represents the mean cytokine concentration from three technical replicates in an experiment (biological replicate). Each row at 0 h and 9 h for each cytokine indicates an experiment with four conditions (thus three cellular experiments were performed) and the supernatants from the same experiment where measured together in the same Luminex® experiment. Thus, each experiment had for each cytokine its own lower limit of detection (LLOD) which was estimated as two standard deviations above background. In case a sample showed for a cytokine a value below the LLOD, this value was replaced by the LLOD in order to still use it for the mean calculation. For each experiment the delta was then calculated between 0 h and 9 h for each cytokine and each condition, respectively.

<table>
<tr><th>Cytokine</th><th>Time</th><th>0 ng/mL LPS</th><th>1 ng/mL LPS</th><th>10 ng/mL LPS</th><th>100 ng/mL LPS</th></tr>
<tr><td rowspan="6">TNF-α</td><td rowspan="3">0h</td><td>0.03</td><td>0.03</td><td>0.05</td><td>0.06</td></tr>
<tr><td>3.07</td><td>2.98</td><td>2.56</td><td>2.61</td></tr>
<tr><td>7.57</td><td>5.45</td><td>5.82</td><td>5.17</td></tr>
<tr><td rowspan="3">9h</td><td>0.02</td><td>370.34</td><td>665.60</td><td>611.29</td></tr>
<tr><td>2.47</td><td>4084.83</td><td>4233.21</td><td>4766.61</td></tr>
<tr><td>5.43</td><td>1568.78</td><td>3337.83</td><td>2891.17</td></tr>
<tr><td rowspan="6">IL-6</td><td rowspan="3">0h</td><td>0.27</td><td>0.23</td><td>0.49</td><td>0.49</td></tr>
<tr><td>0.15</td><td>0.12</td><td>0.29</td><td>0.10</td></tr>
<tr><td>1.79</td><td>1.38</td><td>2.16</td><td>1.83</td></tr>
<tr><td rowspan="3">9h</td><td>0.21</td><td>333.78</td><td>680.30</td><td>645.04</td></tr>
<tr><td>0.07</td><td>2815.06</td><td>3136.78</td><td>3617.18</td></tr>
<tr><td>1.94</td><td>1841.22</td><td>4045.07</td><td>3443.49</td></tr>
<tr><td rowspan="6">IL-8</td><td rowspan="3">0h</td><td>130.83</td><td>132.35</td><td>141.48</td><td>122.94</td></tr>
<tr><td>345.25</td><td>368.63</td><td>294.42</td><td>348.14</td></tr>
<tr><td>346.28</td><td>310.85</td><td>329.66</td><td>283.06</td></tr>
<tr><td rowspan="3">9h</td><td>125.22</td><td>4469.82</td><td>6024.43</td><td>5884.17</td></tr>
<tr><td>320.93</td><td>3901.47</td><td>3800.18</td><td>4023.25</td></tr>
<tr><td>346.62</td><td>15356.50</td><td>17992.53</td><td>21626.91</td></tr>
<tr><td rowspan="6">CXCL1/GROα</td><td rowspan="3">0h</td><td>18.82</td><td>2.30</td><td>16.92</td><td>14.15</td></tr>
<tr><td>60.30</td><td>54.00</td><td>57.88</td><td>46.59</td></tr>
<tr><td>56.20</td><td>56.95</td><td>43.83</td><td>37.34</td></tr>
<tr><td rowspan="3">9h</td><td>15.56</td><td>1573.70</td><td>2218.84</td><td>2268.16</td></tr>
<tr><td>41.74</td><td>4694.84</td><td>5116.34</td><td>5427.13</td></tr>
<tr><td>56.01</td><td>3398.98</td><td>4560.51</td><td>4594.51</td></tr>
<tr><td rowspan="6">CCL2/MCP-1</td><td rowspan="3">0h</td><td>221.72</td><td>207.17</td><td>234.55</td><td>215.23</td></tr>
<tr><td>559.22</td><td>629.80</td><td>476.81</td><td>681.59</td></tr>
<tr><td>786.98</td><td>766.18</td><td>826.02</td><td>627.00</td></tr>
<tr><td rowspan="3">9h</td><td>235.04</td><td>863.95</td><td>886.15</td><td>706.12</td></tr>
<tr><td>609.51</td><td>2755.91</td><td>1680.03</td><td>1832.65</td></tr>
<tr><td>869.16</td><td>3900.06</td><td>4098.99</td><td>2513.09</td></tr>
<tr><td rowspan="6">CXCL2/GROβ</td><td rowspan="3">0h</td><td>1.00</td><td>1.00</td><td>1.00</td><td>1.00</td></tr>
<tr><td>0.30</td><td>0.30</td><td>0.30</td><td>0.30</td></tr>
<tr><td>1.96</td><td>3.29</td><td>1.02</td><td>2.60</td></tr>
<tr><td rowspan="3">9h</td><td>1.00</td><td>92.49</td><td>157.75</td><td>147.17</td></tr>
<tr><td>0.30</td><td>322.51</td><td>386.69</td><td>407.59</td></tr>
<tr><td>1.73</td><td>213.48</td><td>254.87</td><td>249.99</td></tr>
<tr><td rowspan="4">IL-1β</td><td rowspan="3">0h</td><td>0.09</td><td>0.03</td><td>0.07</td><td>0.03</td></tr>
<tr><td>0.85</td><td>1.21</td><td>1.26</td><td>1.32</td></tr>
<tr><td>1.45</td><td>1.45</td><td>1.45</td><td>1.45</td></tr>
<tr><td>9h</td><td>0.03</td><td>10.38</td><td>22.90</td><td>21.67</td></tr>
</table>

		1.26	35.10	19.92	45.63
		1.45	5.32	23.39	49.02
CCL3/MIP-1α	0h	193.89	178.65	195.86	166.60
		166.53	183.02	147.88	133.59
		626.97	532.07	485.55	391.92
	9h	137.35	6967.63	10123.48	7943.75
		73.29	58355.26	49841.69	58355.26
		412.37	21921.42	22751.58	22349.54
CCL4/MIP-1β	0h	458.23	436.92	449.79	404.11
		1058.54	1088.48	1080.44	970.50
		1398.39	1282.39	1262.04	1145.27
	9h	364.47	6777.49	8621.71	7811.11
		729.64	27092.99	27370.28	26175.58
		1245.95	18118.83	21237.78	17429.13
IL-1α	0h	3.75	2.91	2.30	4.49
		1.60	1.60	1.60	2.13
		0.80	1.05	0.86	0.77
	9h	4.33	17.30	21.97	19.84
		1.60	37.95	26.51	37.61
		1.80	15.46	24.09	39.00
IL-4	0h	6.79	4.01	10.07	1.47
		5.34	8.01	4.64	5.26
		12.45	8.30	4.92	5.86
	9h	0.25	40.05	48.70	48.38
		3.00	85.52	80.39	93.42
		7.17	65.45	78.39	75.30
IL-13	0h	25.00	44.13	25.00	25.00
		140.45	129.83	114.05	130.43
		151.56	128.72	99.18	102.06
	9h	42.06	575.64	637.81	592.81
		113.40	1086.40	1168.52	1075.73
		134.07	759.07	1076.68	986.68
IL-12 p70	0h	0.80	0.80	0.80	1.32
		4.63	4.52	6.67	9.80
		3.08	4.37	6.47	0.45
	9h	0.80	53.09	61.66	46.47
		7.26	146.43	127.43	133.11
		9.48	84.20	100.24	97.29
IL-10	0h	1.44	2.58	0.20	0.91
		2.01	2.21	0.99	0.30
		3.16	3.48	1.57	2.84
	9h	0.31	9.36	15.43	14.76
		0.30	18.18	19.04	16.19
		0.79	23.12	27.16	22.03
CCL5/RANTES	0h	0.70	0.70	0.70	0.70
		1.63	1.39	2.36	1.77
		6.11	5.09	4.83	4.27
	9h	0.70	32.17	91.76	100.84
		0.70	91.94	217.54	222.17
		6.47	107.97	229.31	198.00

CXCL5	0h	33.90	29.55	30.32	29.73
		45.47	41.41	47.12	57.94
		41.91	36.28	41.74	37.38
	9h	20.56	253.03	283.14	301.28
		36.85	261.10	295.37	347.09
		47.92	269.18	305.95	342.64

Table S7. Cytokine concentrations (pg/mL) at 0 h and 9 h used to calculate the delta mean values provided in Figure S1 (BMEC monoculture system exposed to LPS). Each value below represents the mean cytokine concentration from three technical replicates in an experiment (biological replicate). Each row at 0 h and 9 h for each cytokine indicates an experiment with four conditions (thus three cellular experiments were performed) and the supernatants from the same experiment where measured together in the same Luminex® experiment. Thus, each experiment had for each cytokine its own lower limit of detection (LLOD) which was estimated as two standard deviations above background. In case a sample showed for a cytokine a value below the LLOD, this value was replaced by the LLOD in order to still use it for the mean calculation. For each experiment the delta was then calculated between 0 h and 9 h for each cytokine and each condition, respectively.

Cytokine	Time	0 ng/mL LPS	1 ng/mL LPS	10 ng/mL LPS	100 ng/mL LPS
TNF-α	0h	0.55	0.51	0.59	0.42
		0.50	0.49	0.47	0.40
		0.02	0.02	0.02	0.02
	9h	0.60	0.85	1.28	2.11
		0.42	0.87	0.96	1.45
		0.02	0.02	0.02	0.02
IL-6	0h	0.73	0.57	0.72	0.60
		0.10	0.10	0.10	0.10
		0.33	0.23	0.35	0.20
	9h	0.67	0.91	1.20	2.07
		0.10	0.12	0.20	0.32
		0.46	0.40	0.35	0.44
IL-8	0h	0.34	0.34	0.51	0.58
		0.10	0.10	0.16	0.10
		0.88	0.19	0.38	0.30
	9h	3.51	4.05	8.75	25.22
		1.84	4.60	3.57	11.90
		0.73	1.39	1.23	2.74
CXCL1/GROα	0h	22.83	37.47	23.58	19.41
		31.71	17.34	31.53	39.20
		10.85	6.53	3.75	7.94
	9h	28.74	24.22	17.51	25.49
		40.12	24.74	43.18	30.34
		2.30	14.34	16.19	4.64
CCL2/MCP-1	0h	6.75	7.23	8.48	5.97
		1.91	2.69	7.95	5.63
		1.40	1.40	1.43	1.40
	9h	8.64	8.42	7.49	10.62
		5.38	2.86	3.62	6.58
		3.72	2.31	3.12	1.40
CXCL2/GROβ	0h	1.02	1.02	1.02	1.17
		0.30	0.30	0.30	0.30

		1.00	1.00	1.00	1.00
	9h	1.02	1.02	1.02	1.02
		0.30	0.30	0.30	0.30
		1.00	1.00	1.00	1.00
IL-1β	0h	1.45	1.45	1.45	1.45
		0.71	0.95	0.97	0.90
		0.03	0.03	0.03	0.03
	9h	1.45	1.45	1.45	1.45
		0.71	0.94	0.95	0.61
		0.03	0.15	0.03	0.03
CCL3/MIP-1α	0h	38.14	36.87	35.82	33.34
		19.14	24.48	39.19	16.00
		78.25	62.44	78.21	71.76
	9h	21.82	33.97	28.95	41.40
		27.17	24.85	23.74	34.61
		73.80	77.45	78.62	69.03
CCL4/MIP-1β	0h	26.06	28.42	31.80	27.33
		29.00	29.00	32.96	42.43
		66.16	22.50	35.69	27.86
	9h	49.39	26.00	54.24	70.27
		38.11	31.23	29.13	45.51
		47.02	30.29	39.37	24.48
IL-1α	0h	0.21	0.16	0.76	0.47
		1.60	1.60	1.60	1.60
		2.30	2.30	2.30	2.31
	9h	1.69	0.70	0.26	2.22
		1.60	1.60	1.60	1.60
		4.14	2.30	3.66	4.33
IL-4	0h	0.90	1.02	1.14	1.40
		3.00	3.00	3.00	4.02
		1.28	3.26	1.56	1.28
	9h	1.62	0.91	1.22	1.40
		3.00	3.05	3.00	6.25
		6.44	3.93	3.17	5.57
IL-13	0h	13.87	10.00	10.00	10.00
		35.70	66.08	61.54	55.52
		25.00	25.00	25.00	29.11
	9h	21.38	12.14	10.00	19.17
		57.37	32.48	76.03	50.90
		25.00	25.00	25.00	25.00
IL-12 p70	0h	3.22	1.22	8.77	0.77
		7.63	2.80	3.08	0.20
		0.80	0.80	0.80	0.80
	9h	7.63	2.23	0.45	3.67
		5.56	6.83	1.68	0.20
		0.80	0.80	0.80	0.80
IL-10	0h	1.82	0.17	0.87	0.60
		1.26	0.41	0.30	0.30
		1.99	0.20	0.88	1.14
	9h	0.17	0.17	0.17	0.17

		1.44	0.30	0.30	0.30
		0.20	0.20	0.20	0.20
CCL5/RANTES	0h	3.76	3.76	3.76	3.76
		0.60	0.60	0.60	0.60
		0.70	0.70	0.70	0.70
	9h	3.76	3.76	3.76	3.76
		0.60	0.60	0.60	0.60
		0.70	0.70	0.70	0.70
CXCL5	0h	0.90	0.40	1.96	1.29
		19.00	19.00	19.00	19.00
		1.13	1.13	4.54	10.16
	9h	1.41	3.52	7.58	11.23
		19.00	19.00	19.00	19.00
		6.21	10.32	7.76	6.91
